# Detection of SARS-CoV-2 antibodies in febrile patients from an endemic region of dengue and chikungunya in Peru

**DOI:** 10.1371/journal.pone.0265820

**Published:** 2022-04-08

**Authors:** Yordi Tarazona-Castro, Lucinda Troyes-Rivera, Johanna Martins-Luna, Felipe Cabellos-Altamirano, Miguel Angel Aguilar-Luis, Hugo Carrillo-Ng, Luis J. del Valle, Sungmin Kym, Sebastian Miranda-Maravi, Wilmer Silva-Caso, Saul Levy-Blitchtein, Juana del Valle-Mendoza

**Affiliations:** 1 School of Medicine, Research Center of the Faculty of Health Sciences, Universidad Peruana de Ciencias Aplicadas, Lima, Peru; 2 Laboratorio de Biologia Molecular, Instituto de Investigación Nutricional, Lima, Peru; 3 Dirección Subregional de Salud de Jaén, Ministerio de Salud, Cajamarca, Peru; 4 Barcelona Research Center for Multiscale Science and Engineering, Departament d’Enginyeria Química, EEBE, Universitat Politècnica de Catalunya (UPC), Barcelona, Spain; 5 Division of Infectious Diseases, Department of Internal Medicine, Chungnam National University School of Medicine, Daejeon, Republic of Korea; Fundacao Oswaldo Cruz, BRAZIL

## Abstract

**Introduction:**

The rapid expansion of the novel SARS-CoV-2 virus has raised serious public health concerns due to the possibility of misdiagnosis in regions where arboviral diseases are endemic. We performed the first study in northern Peru to describe the detection of SARS-CoV-2 IgM antibodies in febrile patients with a suspected diagnosis of dengue and chikungunya fever.

**Materials and methods:**

A consecutive cross-sectional study was performed in febrile patients attending primary healthcare centers from April 2020 through March 2021. Patients enrolled underwent serum sample collection for the molecular and serological detection of DENV and CHIKV. Also, serological detection of IgM antibodies against SARS-CoV-2 was performed.

**Results:**

464 patients were included during the study period, of which (40.51%) were positive for one pathogen, meanwhile (6.90%) presented co-infections between 2 or more pathogens. The majority of patients with monoinfections were positive for SARS-CoV-2 IgM with (73.40%), followed by DENV 18.09% and CHIKV (8.51%). The most frequent co-infection was DENV + SARS-CoV-2 with (65.63%), followed by DENV + CHIKV and DENV + CHIKV + SARS-CoV-2, both with (12.50%). The presence of polyarthralgias in hands (43.75%, p<0.01) and feet (31.25%, p = 0.05) were more frequently reported in patients with CHIKV monoinfection. Also, conjunctivitis was more common in patients positive for SARS-CoV-2 IgM (11.45%, p<0.01). The rest of the symptoms were similar among all the study groups.

**Conclusion:**

SARS-CoV-2 IgM antibodies were frequently detected in acute sera from febrile patients with a clinical suspicion of arboviral disease. The presence of polyarthralgias in hands and feet may be suggestive of CHIKV infection. These results reaffirm the need to consider SARS-CoV-2 infection as a main differential diagnosis of acute febrile illness in arboviruses endemic areas, as well as to consider co-infections between these pathogens.

## Introduction

In December 2019, several cases of pneumonia of unknown etiology emerged in Wuhan, China. The causative agent was identified as SARS-CoV-2 (Severe acute respiratory syndrome Coronavirus 2) [1]. The disease caused by this virus spread rapidly throughout the world and was declared a pandemic by the World Health Organization on March 11, 2020 [2]. Countries located in tropical and subtropical regions were severely affected by the pandemic, while dealing with the burden of other infectious diseases, such as arboviruses including dengue virus (DENV), chikungunya virus (CHIKV), zika virus (ZIKV), among others [3].

The rapid expansion of the novel COVID-19 disease has raised serious public health concerns due to the possibility of misdiagnosis and overlapping diseases in regions where arboviral diseases are endemic [4], given that COVID-19 share common clinical manifestation [5]. For example, concerns of a concurrent outbreak of arboviruses and COVID-19 raised in Brazil, due to the rapid upsurge in cases of both diseases [6]. Moreover, the Pan American Health Organization (PAHO) published an epidemiological update on arboviruses in Latin America, which stated that the pandemic has put much strain on the health systems of this region and decreased their capacity to deal with arboviral diseases [7, 8].

Several reasons have made it difficult to distinguish between COVID-19 and arboviral diseases, as both can be included in the differential diagnosis of undifferentiated acute febrile illness (AFI) [5]. Firstly, both share overlapping characteristics during the early course of the disease such as nonspecific symptoms including fever, headaches, malaise, myalgias and fatigue [4, 6, 9]. The respiratory signs and symptoms may be absent in early COVID-19 and appear later in the course of the disease [9]. Moreover, previous studies in Singapore and Brazil indicate that some cases classified as DENV or CHIKV at initial admission to healthcare centers were later found positive for SARS-CoV-2 [4, 9]. Furthermore, there have been case reports of false-positive dengue results with rapid diagnostic tests in cases of confirmed SARS-CoV-2 infection [10] and cross reactivity between DENV and SARS-CoV-2 has been reported, given that both pathogens may share common antigenic sites [11]. Finally, a diagnosis of COVID-19 or arboviral disease does not exclude the other and co-infections have also been reported [9]. Altogether, these findings make the diagnosis difficult for physicians working in areas where arboviral diseases are endemic. Establishing a correct diagnosis is essential for patient care and infection control, as unsuspected COVID-19 cases presenting as AFI may be managed outside of isolation areas resulting in an increased risk for disease propagation [9].

Few studies have evaluated the impact of the COVID-19 pandemic and arboviral diseases in countries like Brazil and Singapore [4,9]. However, no local estimates in Peru have been reported, a country vastly affected by the COVID-19 pandemic and where arboviruses are endemic. Therefore, we performed the first study in northern Peru to describe the detection SARS-CoV-2 IgM antibodies in febrile patients with a suspected diagnosis of dengue and chikungunya fever.

## Materials and methods

### Study setting

The study was carried out in the province of Jaen located in the department of Cajamarca, Peru. This region is located in northern Peru, sharing boundaries to the north with Ecuador. The province of Jaen has approximately 185 432 inhabitants, with 52% living in urban areas and 48% in rural areas, according to the last national census. This area is endemic for many pathogens responsible for AFI including arboviruses such as dengue virus, chikungunya virus, zika virus and bacterial pathogens such as leptospirosis, rickettsiosis and bartonellosis [12]. On the other hand, this territory was massively affected by the COVID-19 pandemic, classified as a region of epidemiological extreme alert according to the Peruvian ministry of health. More than 20 000 cases of COVID-19 have been reported in the region, according to the epidemiological bulletin released by the Peruvian ministry of health in March 11, 2021 [13].

### Study design and patients

**Fig 1:**
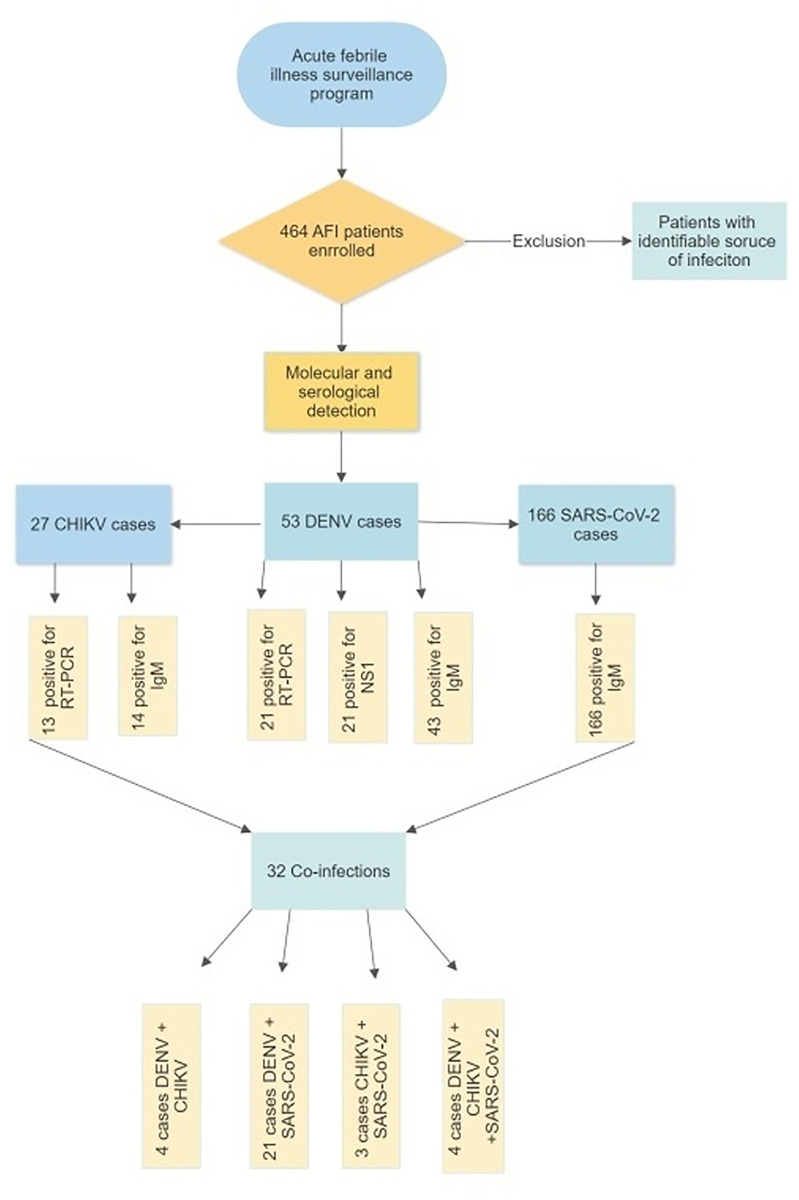
Flowchart of the research methods and study design.

Across-sectional study was performed in patients attending primary healthcare centers in Jaen from April 2020 through March 2021. The study design and research methods are summed up and represented in a flowchart, as shown in Fig 1. This study was carried out jointly with the national surveillance system for the etiological identification of acute febrile syndromes, to provide an aid in the molecular detection of pathogens. Patients are included if they attended outpatient clinics with acute febrile illness (axillary temperature greater than or equal to 38°C in the previous 7 days) along with one or more of the following symptoms: headache, myalgias, arthralgias, retro-ocular pain, lumbar pain, arthritis, nausea, rash, vomiting, conjunctivitis, odynophagia, loss of appetite, among others. Exclusion criteria included patients with an identifiable source of infection, such as acute upper respiratory tract infections, urinary tract infections, among others. A standardized clinical data sheet is filled by attending physicians according to the Peruvian guidelines for the management of AFI and most of the patients are classified as probable dengue cases. Patients who fulfill the inclusion criteria are enrolled and serum samples are collected for the etiological identification of the pathogens. Given that this study was performed as part of the febrile illness national surveillance system, only blood samples were collected. For this reason, the detection of SARS-CoV-2 was not determined by Reverse Transcription Polymerase Chain Reaction (RT-PCR) as nasopharyngeal swab samples were not collected.

### Ethics statement

This study was approved by the ethics and investigation committee of the Regional Hospital of Cajamarca, Peru. The samples were obtained to answer the questions of the present study in the context of the epidemiological / syndromic surveillance program according to the health guidelines of the National Center for Epidemiology, Disease Control and Prevention of the Ministry of Health of Peru. The signing of an informed consent was not required since the study was carried out in a state of emergency by public health mandate in the context of epidemiological surveillance of SARS-CoV-2 and arboviruses.

### Samples and RNA extraction

Patients enrolled underwent serum sample collection for the molecular and serological detection of DENV and CHIKV. Also, serological detection of IgM antibodies against SARS-CoV-2 was performed, given that nasopharyngeal swabs were not available for molecular identification of this virus. One serum sample was obtained for each patient and collected by using Vacuette TUBE Serum Separator Clot Activator (Vacuette, Greiner Bio-One, Kremsmunster, Austria). After collection, all the samples were stored at -80°C. All samples were transported to Lima (Peru) under standardized frozen conditions to perform the assays.

RNA extraction of the samples was performed to carry out molecular detection by RT-PCR for DENV and CHIKV. A total of 200 μL of the serum samples was used for RNA extraction with the High Pure RNA Isolation Kit (Roche Applied Science, Mannheim, Germany), according to the manufacturer’s instructions. Viral RNA obtained after extraction was eluted in 100 μl of nuclease-free water and then processed or stored at -20°C until use.

### Real-time reverse transcriptase PCR amplification for the detection of CHIKV and DENV

Amplification by RT-PCR assay for the detection of CHIKV and DENV were carried out using the primers described by Leparc-Goffart et al. [14], and the PCR conditions were described by Alva-Urcia et al. [15].

### Detection of DENV by NS1 ELISA assay, IgM ELISA assay

The presence of DENV NS1 antigen was detected by Euroimmun ELISA (Euroimmun AG, Lübeck, Germany). DENV IgM antibodies were also detected using Euroimmun ELISA (Euroimmun AG, Lübeck, Germany).

### Detection of CHIKV by IgM ELISA assay

The presence of CHIKV IgM antibodies were also detected using Euroimmun ELISA (Euroimmun AG, Lübeck, Germany).

### Detection of SARS-CoV-2 by IgM ELISA assay

The presence of SARS-CoV-2 IgM antibodies were also detected using Immunodiagnostic; ImmunoDiagnostics Limited Hong Kong Science Park, Sha Tin, Hong Kong. Recombinant nucleocapsid protein (NP) of SARS-CoV-2 is specifically recognized by anti-NP antibodies with a sensitivity and specificity of 88.2% and 92% respectively [16].

In all serological tests, each serum sample was run in duplicate, in accordance with the manufacturer’s instructions.

### Case definition

Cases were defined according to the Peruvian national guidelines for the epidemiological surveillance and diagnosis of arbovirosis [17]. Patients were considered to have dengue fever if they had symptoms and any of the following assays were positive: IgM ELISA and/or NS1 ELISA and/or real time RT-PCR. A case of chikungunya fever was considered when patients had symptoms and a positive IgM ELISA and/or real time RT-PCR. Patients with positive detection of IgM ELISA against SARS-CoV-2 did not have a conclusive COVID-19 diagnosis, however, they were considered to have recent or current infection.

### Statistical analysis

All data were recorded in a database in the Microsoft Excel program. The analysis were performed using the IBM Statistical Package for the Social Sciences (SPSS) version 21.0 software (SPSS, Chicago, IL, USA). Categorical variables were reported as frequencies and percentages. Differences between clinical symptoms and pathogens were evaluated using the Chi-square test. A value of p <0.05 was considered statistically significant. All graphs were made using the GraphPad Prism 9.0.0 program (San Diego, CA, USA).

## Results

**Fig 2:**
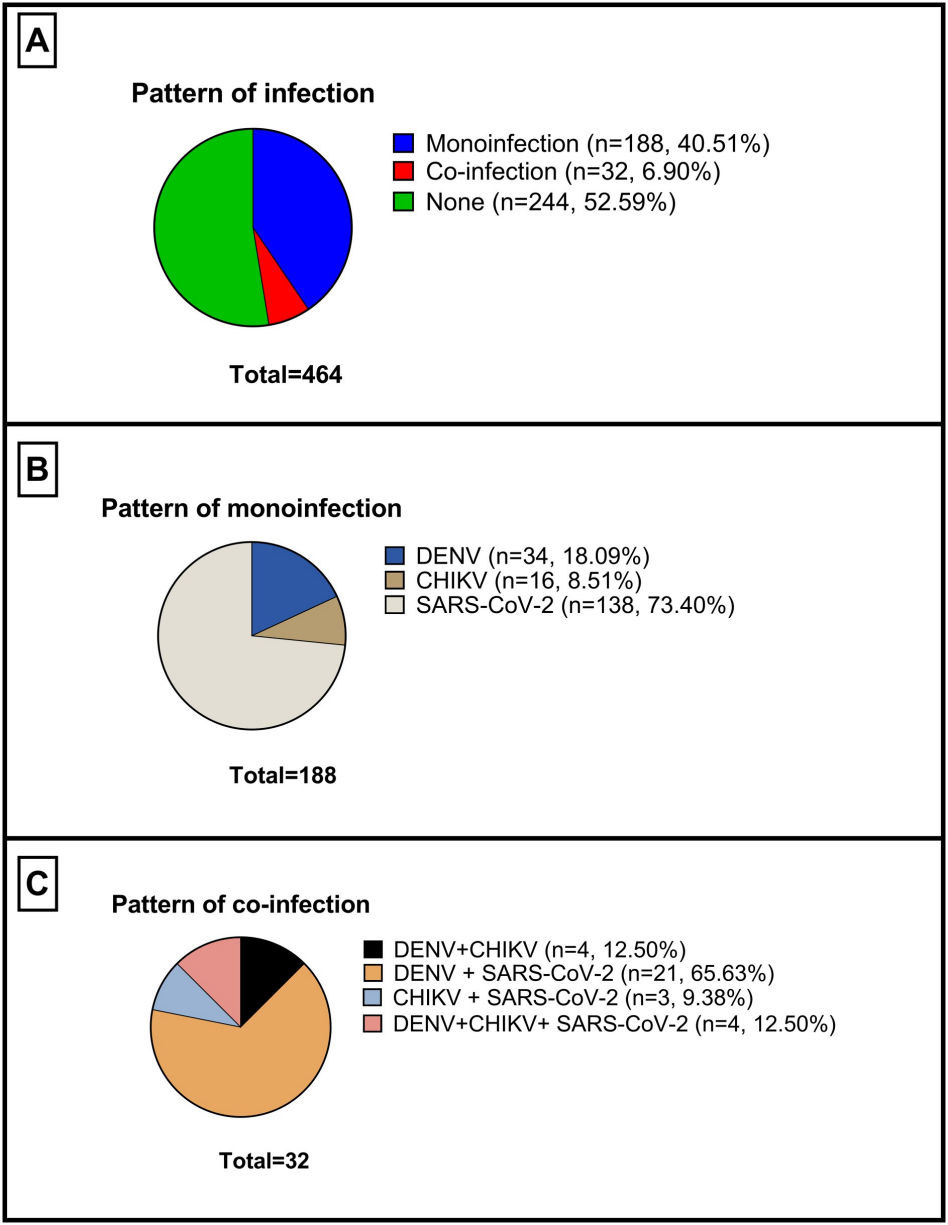
A. Patterns of infection B. Patterns of monoinfection C. Patterns of co-infections.

A total of 464 patients with AFI were enrolled. Fig 2A shows that 188/464 (40.51%) were positive for one pathogen, while 32/464 (6.90%) presented co-infections between 2 or more pathogens. An etiologic agent could not be determined in 244/464 (52.59%) of the cases.

The results of the most frequent pathogens in monoinfections are shown in Fig 2B. In this group the majority of patients tested positive for SARS-CoV-2 IgM with 138/188 (73.40%), followed by DENV 34/188 (18.09%) and CHIKV 16/188 (8.51%). While the results of coinfections are described in Fig 2C. The most frequent co-infection was DENV + SARS-CoV-2 with 21/32 (65.63%), followed by DENV + CHIKV and DENV + CHIKV + SARS-CoV-2, both with 4/32 (12.50%). Finally, CHIKV + SARS-CoV-2 was identified in 3/32 (9.38%) of the co-infections.

Table 1 shows the baseline characteristics of the patients, according to the patterns of infection. It could be observed that the majority were female patients (54.31%) and the predominant age groups were 18–39 years old (42.03%) and 40–50 years old. (32.97%). Table 2 shows the clinical signs and symptoms identified in the patients according to their diagnosis. It can be observed that headaches (82.9%), myalgias (67.03%) and malaise (62.50%) were the predominant symptoms in all patients. We could highlight that polyarthralgias in hands (43.75%, p<0.01) and feet (31.25%, p = 0.05) were more frequently reported in patients with CHIKV monoinfection compared to the other groups. Also, conjunctivitis was more common in patients positive for SARS-CoV-2 IgM (11.45%, p<0.01). The rest of the symptoms were similar among all the study groups.

**Table 1:** Demographic characteristics of the patients according to the pattern of infection.

Characteristics	MONOINFECTIONS				CO-INFECTIONS			
	Total N = 464 (%)	DENV N = 34 (%)	CHIKV N = 16 (%)	SARS-CoV-2 IgM N = 138 (%)	DENV+CHIKV N = 4 (%)	DENV + SARS-CoV-2 IgM N = 21 (%)	CHIKV + SARS-CoV-2 IgM N = 3 (%)	DENV+CHIKV+ SARS-CoV-2 IgM N = 4 (%)
**Age (years)**								
11	12 (2.59)	3 (8.82)	0 (0.00)	1 (0.72)	0 (0.00)	0 (0.00)	0 (0.00)	3 (75.00)
12–17	18 (3.88)	1 (2.94)	0 (0.00)	7 (5.07)	1 (25.00)	1 (4.76)	0 (0.00)	0 (0.00)
18–39	195 (42.03)	17 (50.00)	6 (37.50)	63 (45.65)	3 (75.00)	8 (38.10)	2 (66.67)	1 (25.00)
40–59	153 (32.97)	9 (26.47)	6 (37.50)	38 (27.54)	0 (0.00)	9 (42.85)	1 (33.33)	0 (0.00)
≥ 60	86 (18.53)	4 (11.76)	4 (25.00)	29 (21.01)	0 (0.00)	3 (14.29)	0 (0.00)	0 (0.00)
**Gender**								
Male	212 (45.69)	14 (41.18)	12 (75.00)	58 (42.03)	2 (50.00)	10 (47.62)	0 (0.00)	2 (50.00)
Female	252 (54.31)	20 (58.82)	4 (25.00)	80 (57.97)	2 (50.00)	11 (52.38)	3 (100.00)	2 (50.00)

**Table 2:** Clinical symptoms of patients according the pattern of infection.

	MONOINFECTIONS				CO-INFECTIONS			
	Total N = 464 (%)	DENV N = 34 (%)	CHIKV N = 16 (%)	SARS-CoV-2 IgM N = 138 (%)	DENV+CHIKV N = 4 (%)	DENV + SARS-CoV-2 IgM N = 21 (%)	CHIKV + SARS-CoV-2 IgM N = 3 (%)	DENV+CHIKV+ SARS-CoV-2 IgM N = 4 (%)
**Clinical symptoms**								
Headache	411 (82.9)	30 (88.24)	14 (87.50)	143 (86.14)	4 (100.00)	18 (85.71)	3 (100.00)	2 (50.00)
Myalgias	311 (67.03)	26 (76.47)	10 (62.50)	112 (67.47)	3 (75.00)	14 (66.67)	2 (66.67)	2 (50.00)
Malaise	290 (62.50)	21 (61.76)	12 (75.00)	104 (62.65)	2 (50.00)	16 (76.19)	2 (66.67)	2 (50.00)
Retro-ocular pain	185 (39.87)	16 (47.06)	9 (56.25)	66 (39.76)	2 (50.00)	7 (33.33)	2 (66.67)	0 (0.00)
Lumbar pain	180 (38.79)	11 (32.35)	8 (50.00)	62 (37.35)	2 (50.00)	8 (38.10)	2 (66.67)	1 (25.00)
Joint pain	111 (23.92)	9 (26.47)	5 (31.25)	31 (18.67)	1 (25.00)	2 (9.52)	2 (66.67)	0 (0.00)
Nausea	92 (19.83)	8 (23.53)	2 (12.50)	38 (22.89)	1 (25.00)	8 (38.10)	0 (0.00)	1 (25.00)
Polyarthralgia in hands	87 (18.75)	6 (17.65)	7 (43.75)*	25 (15.06)	1 (25.00)	2 (9.52)	1 (33.33)	0 (0.00)
Polyarthralgia in feet	74 (15.95)	5 (14.71)	5 (31.25)*	24 (14.46)	0 (0.00)	2 (9.52)	1 (33.33)	0 (0.00)
Rash	50 (10.78)	7 (20.59)	1 (6.25)	25 (15.06)	0 (0.00)	5 (25.81)	1 (33.33)	1 (25.00)
Vomiting	44 (9.48)	3 (8.82)	3 (18.75)	16 (9.64)	0 (0.00)	2 (9.52)	0 (0.00)	1 (25.00)
Conjunctivitis	39 (8.41)	1 (2.94)	1 (6.25)	19 (11.45)^†^	0 (0.00)	1 (4.76)	1 (33.33)	0 (0.00)
Loss of apetite	3 (0.65)	0 (0.00)	0 (0.00)	2 (1.20)	0 (0.00)	0 (0.00)	0 (0.00)	0 (0.00)
Odynophagia	2 (0.43)	0 (0.00)	0 (0.00)	1 (0.60)	0 (0.00)	0 (0.00)	0 (0.00)	0 (0.00)
**Warning signs**								
Persistent intense abdominal pain	3 (0.65)	0 (0.00)	1 (6.25)	2 (1.20)	0 (0.00)	0 (0.00)	0 (0.00)	0 (0.00)
Persistent vomiting	1 (0.22)	0 (0.00)	0 (0.00)	1 (0.60)	0 (0.00)	0 (0.00)	0 (0.00)	0 (0.00)
Chest pain	1 (0.22)	0 (0.00)	0 (0.00)	0 (0.0)	0 (0.00)	0 (0.00)	0 (0.00)	0 (0.00)
**Severity signs**								
Arterial blood pressure differential <20 MMHg	1 (0.22)	0 (0.00)	0 (0.00)	0 (0.0)	0 (0.00)	0 (0.00)	0 (0.00)	0 (0.00)

* Chi-square test, CHIKV vs other infections, p<0.05.

^**†**^ Chi-square test, SARS-CoV-2 vs other infections, p<0.05.

**Fig 3:**
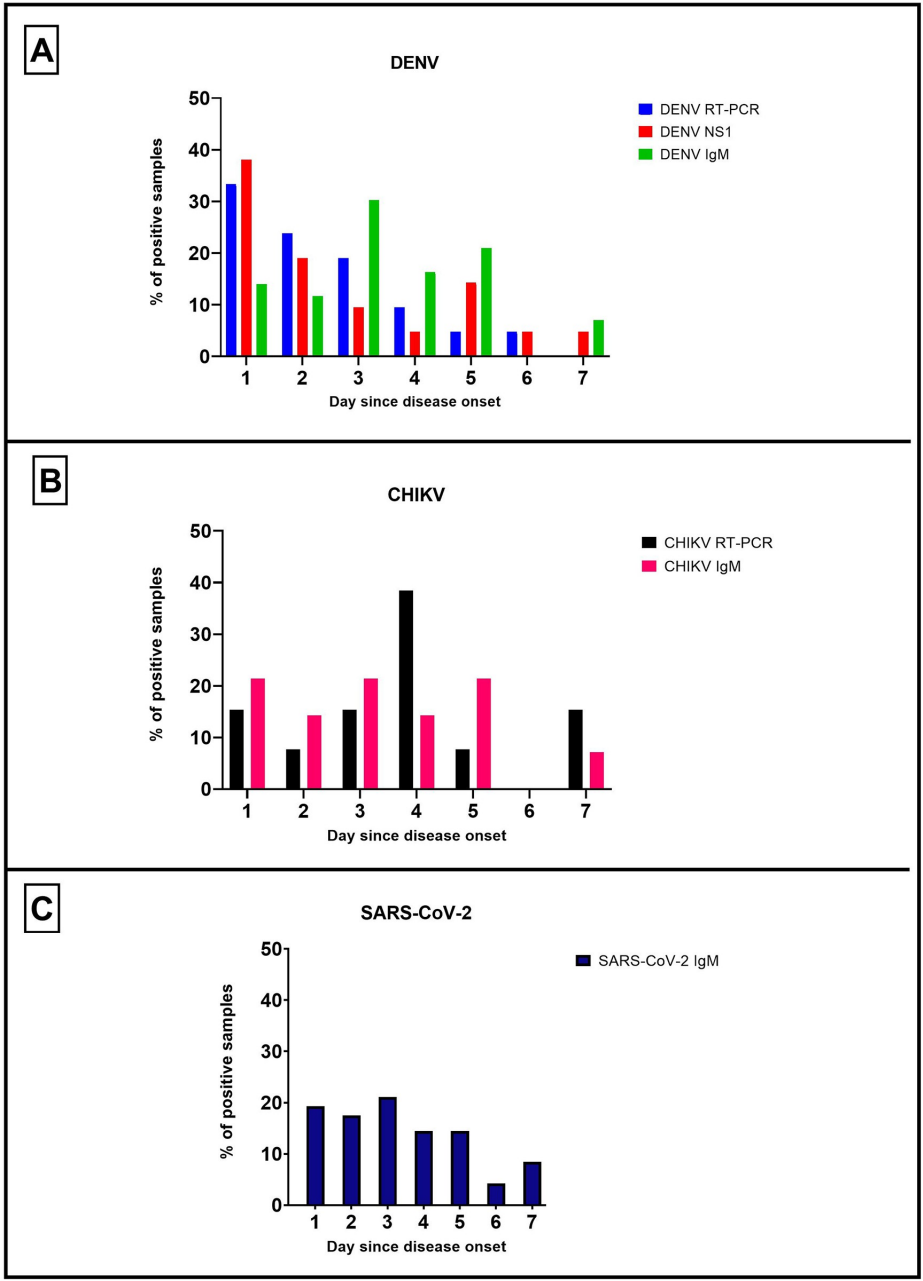
Performance of the different diagnostic assays according to the day of disease.

Fig 3 shows the rate of positive samples of each assay according to the day of disease onset. In the case of DENV (Fig 3A), it can be observed that RT-PCR and NS1 detection performed better in the first 3 days of illness, compared to IgM detection which had a greater detection in days 3–5. In the case of detection of CHIKV (Fig 3B), the diagnosis by RT-PCR was greater in days 3–4, meanwhile IgM detection was similar in the first 5 days. Finally, the detection of IgM against SARS-CoV-2 (Fig 3C) was constant through days 0–5 of disease.

**Fig 4:**
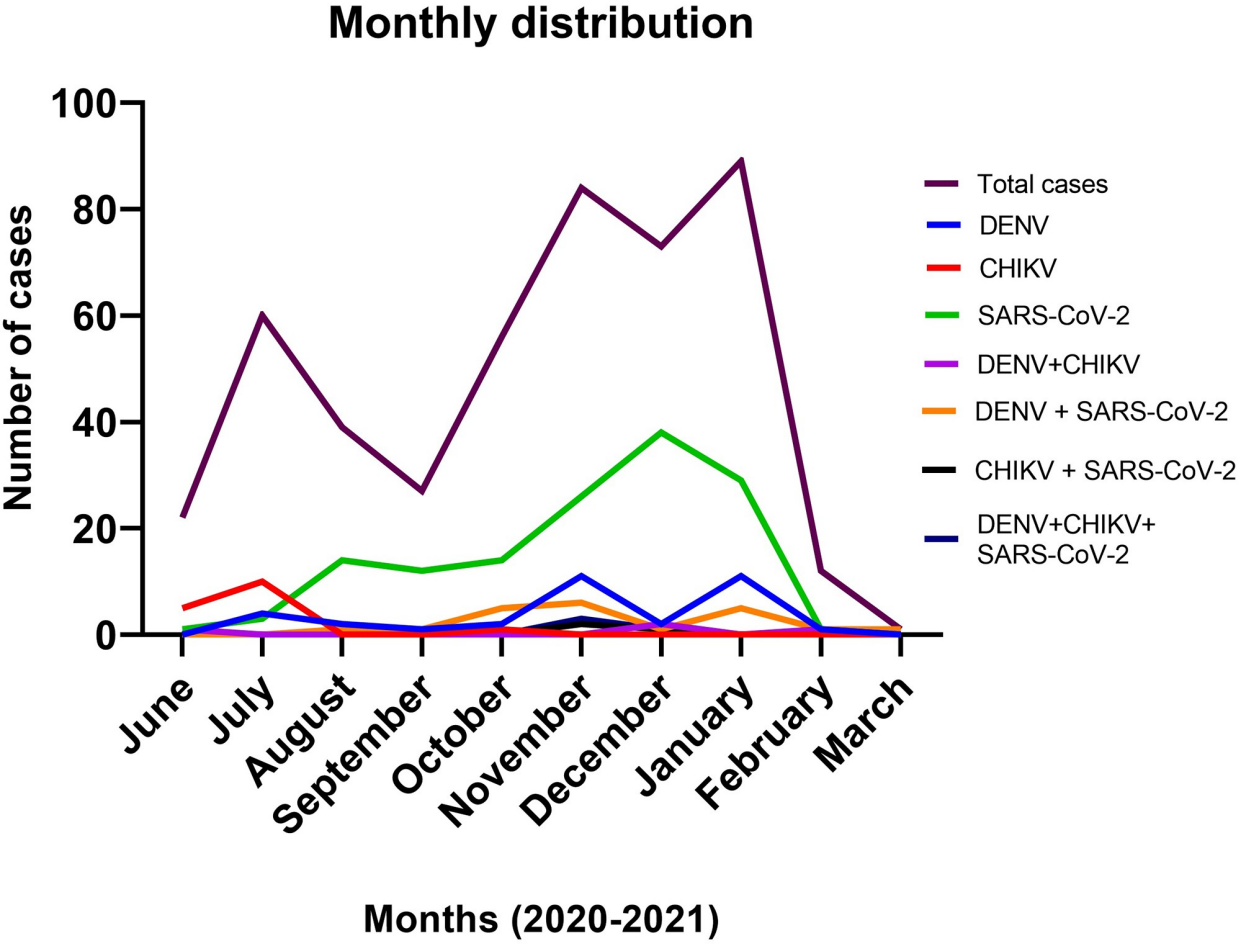
Monthly distribution of the cases according to the pattern of infection.

The monthly distribution of infections by a single and co-infections is shown in Fig 4. It was evidenced that the months with greater cases of AFI were July, October, November and December. Infections by DENV were predominant during the month of November, 2020 and January, 2021; meanwhile CHIKV was more frequent during July, 2020. In the case of SARS-CoV-2, the detection was greater during the last months of 2020, with an increase of cases since October, 2020 through January, 2021.

## Discussion

We enrolled 464 febrile patients in an endemic region of Peru for DENV and CHIKV, in the context of the COVID-19 pandemic. This is the first Peruvian study evaluating the presence of SARS-CoV-2 IgM antibodies in febrile patients from an endemic region of dengue and chikungunya. Despite several advances in diagnostic techniques, the diagnosis of AFI is still challenging in resource-limited countries such as Peru and the etiology cannot always be achieved in these settings [18]. Over the past several years, arthropod-borne viral disease has reemerged in some regions in Peru, among them, dengue virus (DENV) and chikungunya virus (CHIKV) are among the most important [15]. Acute febrile illness is still one of the main causes of morbidity and mortality in low and middle incomes countries [19] and given that specific tests are not always readily available, treatment of patients is based on syndromic management [20, 21]. In the context of the current COVID-19 pandemic, cases of SARS-CoV-2 infection can present as undifferentiated acute febrile illness and managed in the same way as arboviral diseases. A previous study reported that some patients with COVID-19 may go unnoticed in settings with dual outbreaks with dengue infection [9]. The authors propose that a strict triage system may be required for infection prevention and control, in order to mitigate COVID-19 spread.

For the detection of DENV and CHIKV, we used molecular and serological techniques according to the case definition. Meanwhile, for the detection of SARS-CoV-2 only serological tests were used. Molecular identification of SARS-CoV-2 could not be carried out by RT-PCR since the patients included were classified as dengue or chikungunya by the treating physicians and under this criterion, nasopharyngeal swab was not obtained.

We identified that a high proportion of patients were positive for IgM antibodies against SARS-CoV-2. These were more frequent than the DENV and CHIKV cases. This finding is consistent with the results of other studies indicating that in arbovirus endemic regions, SARS-CoV-2 infection should always be considered as an important differential diagnosis in febrile patients. For example, Stringari et al. reported that covert cases of SARS-CoV-2 by identification of IgG antibodies were detected in 2.85% of the total patients in an endemic region for DENV and CHIKV [4]. Another study performed by Wee et al. [9] found that only 8.5% of COVID cases were concurrently tested for DENV, of which 9 patients had positive serology for DENV. Finally, when evaluating the demographic characteristics of the population studied, it was observed that the majority of patients were female and in the 18–59 age group, which is similar to previous studies of AFI and arbovirosis [22]. However, the susceptible population for these diseases is variable among studies [22, 23].

An evaluation of the most frequent symptoms according to the pathogens identified, we could highlight that the most common symptoms among all groups were headaches (82.9%), myalgias (67.03%) and malaise (62.50%). Our results indicate that polyarthralgia in hands and feet were significantly more common in patients with CHIKV and cojunctivitis in patients with positive serology for SARS-CoV-2. These findings could be considered to distinguish between different pathogens. No significant differences in the other symptoms were observed among the different pathogens, which is in agreement with previous studies that report that differentiation between arboviruses is difficult by clinical picture alone [18, 20]. Moreover, in the context of the COVID-19 pandemic, the SARS-CoV-2 virus should also be included in the differential diagnosis in patients with acute undifferentiated febrile illness, given that symptoms may be similar during early course of the disease [4, 5, 6]. Some characteristics symptoms that may aid in the diagnosis are: arthralgias in CHIKV and retro/orbital pain and hemorrhagic diathesis in DENV [24]. However, these symptoms may not always be present and unespecific symptoms predominate during early course of the disease. In our study, patients were recruited from outpatient health centers, therefore, they were more likely to present with mild symptoms and no severe cases were reported.

Regarding the severity of the disease, it has been described that arbovirus and SARS-CoV-2 coinfections do not present with more severe symptoms compared to monoinfections. Therefore, patients have a favorable clinical course during the duration of the disease [4, 25]. Nor has it been described that SARS-CoV-2 co-infections with other respiratory pathogens imply that the disease develops in a more severe way [26]. Our study reports the presence of alarm signs in two patients with CHIKV and SARS-CoV-2 monoinfection.

When evaluating the seasonal pattern of each pathogen, we could observe that the majority of AFI cases occurred during the last months of 2020; however, concurrent outbreaks were not identified. It was observed that most cases of CHIKV were identified during July, 2020; on the other hand, DENV cases occurred during November, 2020 and January, 2021. The identification of SARS-CoV-2 cases was constant through the 2020 year, with a peak in December. It has been reported that dual outbreaks between SARS-CoV-2 and DENV have occurred in other regions, with cases of both pathogens coinciding in a specific time lapse [20, 21]. However, it has also been observed a seasonal decrease in DENV cases with an increasing slope of SARS-CoV-2 [27], similar to what occurred in our study during the last months of 2020. It has been proposed that SARS-CoV-2 cases may be displacing the usual cases in this endemic zone for DENV and CHIKV, which could be explained by a phenomenom called viral interference, a process in which a high pathogenic virus inhibits the entry and replication of another virus [21, 27]. Our study adds valuable information regarding the relationship between arboviruses and SARS-CoV-2, as few studies have been published as indicated by a previous systematic review [3].

We used accurate diagnostic tests for the identification of the pathogens studied and performed and evaluation of their performance according to the day since disease onset. In the case of diagnostic methods for DENV we could observe that NS1 detection and RT-PCR performed better during the initial days of disease, which may be correlated to the early viremia observed in this disease [28]. On the other hand, the detection of IgM antibodies was greater on days 3, 4 and 5 since disease onset, which is the period for humoral response to be achieved. This is in agreement with previous studies that indicate that IgM antibodies against DENV can appear as early as the 3–5 day since disease onset [28–30]. In the case of diagnostic assays for CHIKV, we could observe that performance of RT-PCR was constant through days 1–7, which could indicate a persistent viremia compared to DENV, as reported previously [31–33]. In the case of IgM antibodies detection, we could observe a similar detection through days 1–5, possibly due to early antibodies production against this pathogen [34].

There are four major types of serological diagnostic tests [35]. In the current study we used an ELISA-based assay for the detection of IgM antibodies against SARS-CoV-2. Detection was achieved in patients through days 1–7 since disease onset, with a peak on days 2 and 3. Previous studies suggest that seroconversion may occur days after the viral load has peaked [34, 35], consequently serological tests would be less effective in the early stages of infection. For example, the median time for the detection of IgM antibodies was reported to be 5 days, ranging from 3–6 days, according to Guo et al [36]. However, some limitations arise from using this assay for the diagnosis of SARS-CoV-2. For example, previous cross-reactivity has been reported with other coronaviruses [35], as well as with DENV [10]. Also, negative results may be obtained if the patients present during the early days of the disease when antibodies against SARS-CoV-2 have not been formed yet, so collection of paired sera from the subjects may be advisable in some cases. Nonetheless, the use of serological tests for the diagnosis of SARS-CoV-2 was a useful tool during the peak of the pandemic, particularly in resource-limited settings where molecular tests were not available.

The most important limitation of this study is that molecular detection of SARS-CoV-2 could not be performed, and diagnosis was based on serological assays. The possibility of cross-reaction in serological tests for Dengue and SARS-CoV-2 has also been described by Lustig et al. [37];however, this study reported cross-reactivity with tests that detect IgA and IgG against the S protein of SARS-CoV-2. In the case of our study, we used an ELISA-based assay for the detection of IgM antibodies against the nucleocapsid protein. Nonetheless, robust independent post-marketing evaluations are needed to confirm manufacturers’ performance claims [37]. On the other hand, the detection of IgM limits the possibility of diagnosis in the first days of the disease, so no cases may have been identified in the early onset of the disease. Another limitation is that symptoms and days of disease are self-reported by the patients and registered in a clinical sheet by attending physicians which could induce heterogeneity in the data. Another important limitation is that we did not evaluate the presence of Zika virus infection among the patients enrolled in the study. This virus may have various overlapping symptoms with the other pathogens studied, particularly ophthalmic manifestations [38]. Finally, these results may not be extrapolated to other settings, given that it was carried out in a specific geographical area and temporal period.

In conclusion we found a large prevalence of SARS-CoV-2 IgM antibodies in acute sera from febrile patients with a clinical suspicion of arboviral disease. Co-infection between DENV and SARS-CoV-2 were also frequent. Symptoms among the different study groups were similar, however, the presence of polyarthralgias in hands and feet may be suggestive of CHIKV infection. These results reaffirm the need to consider SARS-CoV-2 infection as a main differential diagnosis of acute febrile illness in arboviruses endemic areas, as well as to consider co-infections between these pathogens Accurate and timely diagnostic assays, in combination with suggestive clinical symptoms are required to correctly distinguish these pathogens.
